# Case Report: Progressive Asymmetric Parkinsonism Secondary to CADASIL Without Dementia

**DOI:** 10.3389/fneur.2021.760164

**Published:** 2022-01-10

**Authors:** Weihang Guo, Baolei Xu, Hong Sun, Jinghong Ma, ShanShan Mei, Jingrong Zeng, Junyan Sun, Erhe Xu

**Affiliations:** ^1^Department of Neurobiology, Neurology and Geriatrics, Xuanwu Hospital of Capital Medical University, Beijing Institute of Geriatrics, Beijing, China; ^2^Clinical Center for Parkinson's Disease, Capital Medical University, Beijing, China; ^3^Key Laboratory for Neurodegenerative Disease of the Ministry of Education, Beijing Key Laboratory for Parkinson's Disease, Parkinson's Disease Center of Beijing Institute for Brain Disorders, Beijing, China; ^4^National Clinical Research Center for Geriatric Disorders, Beijing, China

**Keywords:** parkinsonism, cerebral autosomal dominant arteriopathy with subcortical infarction and leukoencephalopathy, cognition, rapid eye movement sleep behavior disorder (RBD), case report

## Abstract

Parkinsonism is a rare phenotype of cerebral autosomal dominant arteriopathy with subcortical infarction and leukoencephalopathy (CADASIL), all of which involve cognitive decline. Normal cognition has not been reported in previous disease studies. Here we report the case of a 60-year-old female patient with a 2-year history of progressive asymmetric parkinsonism. On examination, she showed severe parkinsonism featuring bradykinesia and axial and limb rigidity with preserved cognition. Magnetic resonance imaging (MRI) revealed white matter hyperintensity in the external capsule and periventricular region. Dopaminergic response was limited. A missense mutation c.1630C>T (p.R544C) on the *NOTCH3* gene was identified on whole-exome sequencing, which confirmed the diagnosis of vascular parkinsonism secondary to CADASIL. A diagnosis of CADASIL should be considered in asymmetric parkinsonism without dementia. Characteristic MRI findings support the diagnosis.

## Introduction

Cerebral autosomal dominant arteriopathy with subcortical infarction and leukoencephalopathy (CADASIL) is an adult-onset disorder caused by *NOTCH3* gene mutations. CADASIL is characterized by young or middle-aged adult onset, migraine with aura, stroke, psychiatric symptoms, and cognitive impairment. Recent reports have extended the clinical spectrum associated with CADASIL (1–3) and state that parkinsonism is a late, not rare, feature of CADASIL. Parkinsonism refers to bradykinesia combined with either resting tremors, rigidity, or both. However, progressive asymmetric vascular parkinsonism with normal cognition secondary to CADASIL has not been previously reported.

## Case Description

A 60-year-old female presented with a 2-year history of progressive worsening of left upper limb tremors, sleep disturbance, and urinary incontinence. She was diagnosed with Parkinson's disease; however, dopaminergic therapy was ineffective. Twenty years prior to our clinical evaluation, the patient exhibited symptoms typical of rapid eye movement sleep behavior disorder (RBD), such as dream-enacting behavior (shouting and punching related to unpleasant dreams). Two years before presentation, she developed a tremor in her left upper extremity. The tremor gradually worsened and the patient complained of clumsiness and bradykinesia of the left limb 1 year later. Two months before we performed clinical evaluation, the patient reported urinary urgency, frequency, and incontinence without orthostatic dizziness, falls, hyposmia, or visual hallucinations. Her parkinsonian symptoms responded poorly to L-dopa therapy (125 mg/day). The patient denied experiencing migraines, dementia, symptoms of stroke, or psychosis, even as her disease progressed. She had a history of supraventricular tachycardia treated with radiofrequency ablation 11 years previously. Hypertension, diabetes, hyperlipidemia, history of smoking or drinking alcohol, and other risk factors for stroke were not present. No other family members had a similar medical history.

Positive parkinsonian findings on the patient's physical examination included a mask-like face, postural tremor of the left hand, axial and left limb rigidity, and akinesia. Negative findings included normal vertical eye movements, negative pull test, and negative Babinski sign bilaterally. Blood pressure (BP) measurements revealed that the patient didn't have orthostatic hypotension (systolic/diastolic BP: 115/68 mmHg in recumbent and 112/60 mmHg in standing position). The Unified Parkinson's Disease Rating Scale (UPDRS) score was 66 points (UPDRSIII score was 36 points during off-period). Improvement rate was 25% for the levodopa challenge test (levodopa 200 mg plus 50 mg decarboxylase inhibitor). Neuropsychological test results were normal. The Mini-Mental State Examination score was 30 points and the Montreal Cognitive Assessment score was 27 points. The residual urine volume measurement was normal.

**Figure 1:**
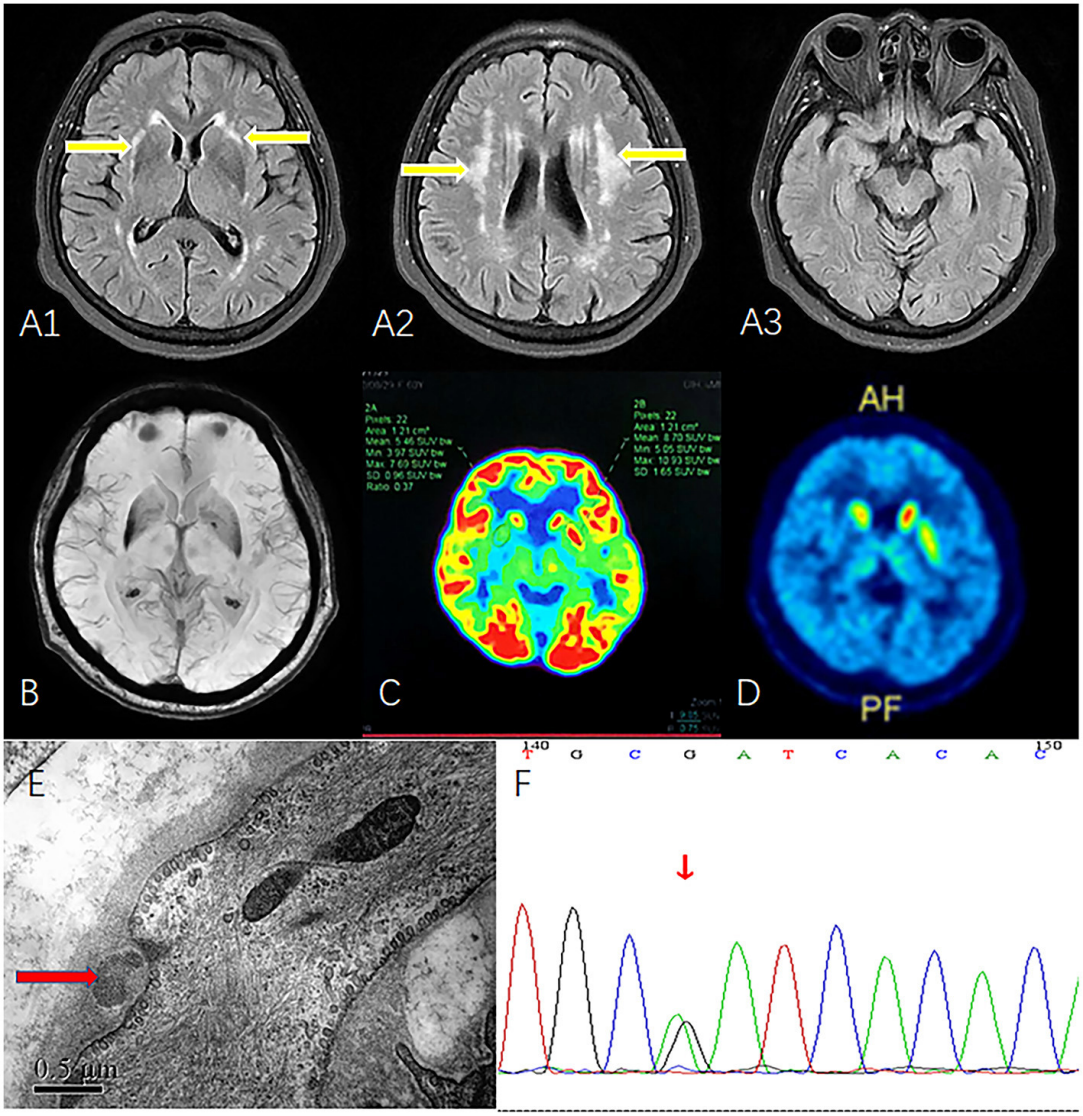
MRI, FDG-PET, VMAT2-PET, skin biopsy, and Sanger sequencing. **(A1)** FLAIR image shows white matter hyperintensity of the external capsule (yellow arrow). **(A2)** FLAIR image shows periventricular white matter hyperintensity (yellow arrow). **(A3)** Bilateral temporal poles show normal signal. **(B)** Flair image revealed no clearly cerebellar atrophy. **(C)** FDG-PET reveals hypometabolism in the right putamen. **(D)** VMAT2 indicates low-intensity signals in right putamen. **(E)** Skin biopsy shows granular osmiophilic material near the basement membrane of arteriolar smooth muscle cells (red arrow). **(F)** Sanger sequencing confirmed the mutation c. 1630C>T (p.R544C). MRI, magnetic resonance imaging; FDG-PET, fluorodeoxyglucose-positron emission tomography; FLAIR, fluid-attenuated inversion recovery; VMAT2, vesicular monoamine transporter type 2.

Brain magnetic resonance imaging (MRI) showed fluid-attenuated inversion recovery (FLAIR) hyperintensity throughout the white matter of both the external capsule and the periventricular region. However, high signals were not observed on bilateral temporal poles (Figure 1A). Flair image revealed no clearly cerebellar atrophy (Figure 1B). Fluorodeoxyglucose (FDG)-positron emission tomography (PET)/computed tomography showed hypometabolism in the bilateral putamen, especially on the right side (Figure 1C). Vesicular monoamine transporter type 2 (VMAT2) imaging with PET also indicated a low signal in the right putamen (Figure 1D). Skin biopsy showed the presence of granular osmiophilic material (Figure 1E), which confirmed the diagnosis of CADASIL. A missense mutation c. 1630C>T (p.R544C) on the *NOTCH3* gene was identified on whole-exome sequencing and verified by Sanger sequencing (Figure 1F). At this point, the patient was given a final diagnosis of CADASIL with vascular parkinsonism. We treated the patient with aspirin (100 mg/day), and although there was no significant improvement in patient's symptoms during the 6-month follow-up period.

## Discussion

This is the first reported case of progressive asymmetric parkinsonism secondary to CADASIL without dementia, confirmed by skin biopsy and genetic testing. We were unable to access clinical information on the patient's family members to investigate the possibility of familial inheritance.

Our patient presented with gradually progressive asymmetric parkinsonism. Motor symptoms included tremors, bradykinesia, and rigidity. Non-motor symptoms that supported the diagnosis of parkinsonism included RBD and urinary urgency, frequency, and incontinence. Urinary incontinence within 5 years is a red flag for idiopathic Parkinson's disease. FDG-PET and VMAT2 results confirmed that the external capsule hyperintensity involved the putamen. The lesion in the right putamen may explain the contralateral motor symptoms. We considered that the hypometabolism and atrophy of the putamen were caused by the adjacent vascular lesion, which supported the diagnosis of vascular parkinsonism. FLAIR revealed external capsule hyperintensity and white matter degeneration, suggesting the diagnosis of CADASIL. Skin biopsy and whole exome sequencing confirmed the diagnosis of CADASIL.

The patient had normal residual urine volume; thus, we considered that urinary incontinence was caused by the white matter lesion of CADASIL, which was reported in previous study (3). Further, her BPs in recumbent and standing positions were normal. We believe that the patient does not have autonomic failure. Besides, the incidence of both MSA and CADASIL is low (MSA: 0.6/100,000; CADASIL: 4.6/100,000) (4, 5). We cannot completely exclude the possibility of comorbidity with both, although the possibility is small.

Previous studies reported that CADASIL patients with a clinical phenotype of parkinsonism were characterized by progressive supranuclear palsy, lower body parkinsonism, and asymmetric parkinsonism. We summarized previous reported in Table 1. The age of onset in these patients was often >53 years and they usually presented with cognitive impairment and stroke. All cases presented a limited levodopa response (3, 6–8). Our patient presented with isolated asymmetric parkinsonism and RBD without cognitive decline. Higher educational levels and phenotypic heterogeneity caused by gene-environment interactions may account for normal patient cognition. Limited levodopa response may be due to disease involvement of the putamen.

**Table 1:** Clinical manifestation of previous reported CADASIL with parkinsonism.

**References**	**Sex/age**	**Age of onset**	**Exome**	**Amino acid change**	**Symptoms**	**Other symptoms**	**Family history**	**Other phenotypes of other family members**
Van Gerpen et al. (6)	F/60y	54	NA	NA	Symmetric PDS	Dementia/ stroke	Y	Dementia, stroke
Wegner et al. (3)	M/55y	53	NA	NA	Asymmetric PDS/ upward gaze palsy	Dementia/ depression/ urinary incontinence	N	NA
Valenti et al. (7)	M/64y	60	24	C1315T	Symmetric PDS	Dementia	Y	PDS/stroke/ Dementia
Ragno et al. (8)	4M1F/63.0 ± 9.3y	61 ± 8	19	A1006C	1 asymmetric PDS/ 4 symmetric PDS	Dementia	Y	Dementia, stroke
Erro et al. (1)	M/76y	72	3	R103X	Asymmetric PDS/ upgaze supranuclear palsy	Dementia	Y	Stroke
Ragno et al. (2)	M/75y	75	19	A1006C	Asymmetric PDS	MCI	N	NA
Our case	F/60y	58	11	R544C	Asymmetric PDS/RBD	Urinary incontinence	N	NA

*F, female; M, male; PDS, parkinsonian syndrome; RBD, rapid-eye-movement behavior disorder; MCI, mild cognitive impairment; Y, yes; N, no; NA, no information available*.

The spectrum of CADASIL clinical phenotypes was various. Typical course, such as previous stroke and migraine attack, were absent in this case. It has been previously reported in the literature that *NOTCH3* R544C mutation shows atypical clinical manifestations. And this mutation is associated with fewer temporal pole lesions, older age of onset, cognitive dysfunction, and is less associated with migraine (9, 10). Besides, Notch3 mutations have been found in primary Parkinson's disease in a previous study (11), in which 13 of 139 patients with Parkinson's disease had rare *NOTCH3* mutations. These mutations were associated with the number of paraventricular white matter hyperintensities. While the mutation in this case is the reported causative mutation *NOTCH3* R544C, the present case is a confirmed CADASIL with parkinsonism. Parkinson's disease and CADASIL may have a comorbid phenotype mediated by NOTCH3 mutations.

There are some limitations in this article. At first, the other family members of the patient did not perform gene test and brain MRI, although the physical examination and past history were normal. Subclinical symptom may be found in the progressive clinical examination. Secondly, we cannot completely exclude the possibility of comorbidity between MSA and CADASIL. Urodynic study should be performed to identify the urinary incontinence. A longer follow-up period is necessary.

Careful consideration is required in the diagnosis of patients with late-onset progressive asymmetric parkinsonism. Clinicians should consider CADASIL as a differential diagnosis of parkinsonism phenotype, particularly when the patient has past history of premature stroke or progressive cognitive impairment and neuroimaging demonstrates multiple subcortical infarcts. Evaluation for characteristic white matter hyperintensity in the external capsule and temporal pole should be a focus in future clinical diagnostic testing. Genetic testing and skin biopsy should also be done to confirm the diagnosis.

## Data Availability Statement

The original contributions presented in the study are included in the article/supplementary material, further inquiries can be directed to the corresponding author.

## Ethics Statement

The studies involving human participants were reviewed and approved by the Medical Research Ethics Committee at Xuanwu Hospital. The patients/participants provided their written informed consent to participate in this study. Written informed consent was obtained from the patient for publication of this case report and any accompanying images. A copy of the written consent is available for review upon request.

## Author Contributions

WG analyzed and interpreted the data and wrote the manuscript. BX, HS, JM, SM, JZ, and JS analyzed, interpreted the data, and revised the manuscript. EX designed and conceptualized the study, interpreted the data, and revised the manuscript. All authors contributed to the article and approved the submitted version.

## Funding

The National Key R&D Program of China (2017YFC1310200) provided financial support to conduct this research, including the study design, collection, analysis, interpretation of data, and manuscript writing.

## Conflict of Interest

The authors declare that the research was conducted in the absence of any commercial or financial relationships that could be construed as a potential conflict of interest.

## Publisher's Note

All claims expressed in this article are solely those of the authors and do not necessarily represent those of their affiliated organizations, or those of the publisher, the editors and the reviewers. Any product that may be evaluated in this article, or claim that may be made by its manufacturer, is not guaranteed or endorsed by the publisher.

## Abbreviations

CADASIL: cerebral autosomal dominant arteriopathy with subcortical infarction and leukoencephalopathy
MRI: magnetic resonance imaging
RBD: rapid eye movement sleep behavior disorder
UPDRS: Unified Parkinson's Disease Rating Scale
FLAIR: fluid-attenuated inversion recovery
FDG: fluorodeoxyglucose
PET: positron emission tomography
VMAT2: Vesicular monoamine transporter type 2
MSA: multiple system atrophy.
